# The Noisy Ones

**Published:** 1892-06

**Authors:** 


ARTICLE VI.

### THE NOISY ONES.

Comparisons are always odious. He, who, in private matters, calls himself great, and his neighbor small, is voted a braggart or a bumpkin, yet in public it seems a man may violate all the canons of self respect in his self adulation, and yet we tolerate him. True, he will adopt a round-about phraseology—he will not tell you directly that he is the greatest dentist alive, but he will tell you he was a student at such and such a school,—he will dilate to you on the absolute superiority of this school, and gently hint he was not the worst student at that school. That a man should love his land, his home, and his school, is well. It is well that he should regard its good points with satisfaction, but he should not close his eyes to its failings. He should learn all there is to learn at home, but he should not rest here. As the great thinker, Goethe, let Wilhelm Meister follow his "Lehrjahre" spent in the home by "Wanderjahre" occupied by travels afar, so an ideal student would seek further knowledge in other lands, and at other schools. At Paris, at Vienna, at Berlin, the English Medical student seeks to extend his knowledge and experience, in the same way that Americans and others garner in what we have to teach in our schools at home. So with dental students, after learning what they can at home, they often go to other schools and learn much there. It is well it should be so, and none but the silliest of silly men, would ever dream of thinking that a student, in so doing, cast a slur on his school at home. If an English student, he remains an English student, who has but added a little to his knowledge, and if an American, he remains an American. As Englishmen, we are proud of our schools, but we hope we may not fall in the error of the braggadocio, and more especially, that we may never seek to elevate our own schools by decrying others. If we err, may some kind friend check us. And as we hope we may not so sin ourselves, it is also our wish to credit others with the same intentions. From a variety of circumstances, but more especially on account of superior educational advantages, the United States and England have become, for the time, the chief teaching centres of Dental Surgery. Of England we know, and of the States, two interesting papers have recently appeared, the one pessimistic, the other optimistic, treating of the matter. From the first, that of Mr. Bentley, of Chicago, one rises with the impression, that whatever else the schools may be, they are directly antagonistic to the interests of the Profession at large. Why? Because many have been started, not with the idea of teaching, but simply with that of making money. They, therefore, do not much care about the quality of the man they turn out, so long as there is quantity. And not only is the Profession overcrowded with inferior men, but the very schools themselves enter into competition with their own graduates, and this not on equal terms, for, whilst the graduate is debarred by professional etiquette from advertising, the school does so *ad lib.* For this state of things, he sees a remedy in State Examining Boards, an opinion, however, which meets with a direct negative from Dr. W. C. Barrett, in the second paper referred to, who is perfectly satisfied with the present condition of things. Just as on this point we have opinions utterly divergent, so on the question of American, and may be of English Schools. On the one hand, we have men crying themselves hoarse in self-adulation of American, and may be of English Dentistry, so on the other, we have men equally noisy in running down these very things. How is it possible to reconcile such opposite opinions? Briefly, we think this may be done by seeking the opinions of the silent members of the Profession, of those men of sufficient strength of mind to notice the faults in that they hold most dear. As it is not fair to take a braggart at his own estimate, so neither is it just to blame a Profession for the sins of the few, nor to think that the bulk of this profession have part or parcel in the quack methods and practices with which these few do their best to saddle the whole. True, it is difficult to ignore these noisy members, and far more so when distance prevents our hearing and seeing for ourselves that this verbage represents but a minority in councils and votes on professional matters. Still let us remember:—

"Bean-pods are noisest when dry,
And you always wink with your weakest eye."

—*Ed. in British Journal.*

---

ARTICLE VII.

